# Official Websites Providing Information on COVID-19 Vaccination: Readability and Content Analysis

**DOI:** 10.2196/34003

**Published:** 2022-03-15

**Authors:** Andy Wai Kan Yeung, Thomas Wochele-Thoma, Fabian Eibensteiner, Elisabeth Klager, Mojca Hribersek, Emil D Parvanov, Dalibor Hrg, Sabine Völkl-Kernstock, Maria Kletecka-Pulker, Eva Schaden, Harald Willschke, Atanas G Atanasov

**Affiliations:** 1 Oral and Maxillofacial Radiology, Applied Oral Sciences and Community Dental Care Faculty of Dentistry The University of Hong Kong Hong Kong China; 2 Ludwig Boltzmann Institute for Digital Health and Patient Safety Vienna Austria; 3 Division of Pediatric Nephrology and Gastroenterology Department of Pediatrics and Adolescent Medicine, Comprehensive Center for Pediatrics Medical University of Vienna Vienna Austria; 4 Department of Translational Stem Cell Biology Research Institute of the Medical University of Varna Varna Bulgaria; 5 Medical University of Innsbruck Innsbruck Austria; 6 Department of Computer Science University of Innsbruck Innsbruck Austria; 7 Graz University of Technology Graz Austria; 8 Institute for Ethics and Law in Medicine University of Vienna Vienna Austria; 9 Department of Anaesthesia, Intensive Care Medicine and Pain Medicine Medical University of Vienna Vienna Austria; 10 Institute of Genetics and Animal Biotechnology Polish Academy of Sciences Jastrzebiec Poland

**Keywords:** COVID-19, coronavirus, SARS-CoV-2, vaccine, readability, content quality, online health information, side effect, public health, medicine, quality, perception

## Abstract

**Background:**

Online information on COVID-19 vaccination may influence people’s perception and willingness to be vaccinated. Official websites of vaccination programs have not been systematically assessed before.

**Objective:**

This study aims to assess and compare the readability and content quality of web-based information on COVID-19 vaccination posted on official/governmental websites. Furthermore, the relationship between evaluated website parameters and country vaccination rates were calculated.

**Methods:**

By referring to an open data set hosted at Our World in Data, the 58 countries/regions with the highest total vaccination count as of July 8, 2021, were identified. Together with the websites from the World Health Organization and European Union, a total of 60 vaccination campaign websites were targeted. The “frequently asked questions” or “questions and answers” section of the websites were evaluated in terms of readability (Flesch Reading Ease score and Flesch-Kincaid Grade Level), quality (Health On the Net Foundation code [HONcode] certification and Quality Evaluation Scoring Tool), and content stating vaccination duration of protection and potential side effects.

**Results:**

In terms of readability, the Flesch Reading Ease score of the vaccination frequently asked questions websites ranged between 11.2 and 69.5, with a mean of 40.9 (SD 13.2). Meanwhile, the Flesch-Kincaid Grade Level ranged between 6.5 and 17.6, with a mean of 12.1 (SD 2.8). In terms of quality, only 2 websites were HONcode certified, and the Quality Evaluation Scoring Tool score of the websites ranged between 7 and 20, with a mean of 15.3 (SD 3.1). Half of the websites (25/50) did not present a publication date or date of the last update. Regarding the duration of protection offered by the vaccines, 46% (23/50) of the websites stated that they do not know, and another 40% (20/50) did not address it. Five side effects of the vaccinations were most frequently mentioned, namely, fever/chill (41/50, 82%), various injection site discomfort events (eg, swelling, redness, or pain; 39/50, 78%), headache (36/50, 72%), fatigue (33/50, 66%), and muscle/joint pain (31/50, 62%).

**Conclusions:**

In general, the content quality of most of the evaluated websites was good, but HONcode certification should be considered, content should be written in a more readable manner, and a publication date or date of the last update should be presented.

## Introduction

The COVID-19 pandemic has affected the global population since the end of 2019 and has no signs of resolution as of late 2021. Its fatality rate varied by population and viral strains, with earliest rates reported to be 2.3% in China and 7.2% in Italy [1]. A recent meta-analysis computed an overall infection fatality rate of 0.68% [2]. The COVID-19 morbidity has incurred substantial economic burden, and a study examining the matter estimated that the mean direct medical costs (cost incurred by patient care in the hospital; eg, bed service, consultation, nursing, imaging, medicine, and laboratory) were US $3755 and indirect costs (including income loss due to premature death and productivity loss due to hospitalization and during recovery) were US $11,634 per person [3]. Another study reported that patients with COVID-19 stayed in the hospital for 5 days on average, and the incurred median hospital costs were US $12,046 [4]. In short, the COVID-19 pandemic has caused a heavy burden on society and public health on a global scale. Being a powerful tool to counteract infection, currently available vaccines against COVID-19 may substantially aid mitigation of this pandemic.

Meanwhile, vaccination against COVID-19 became possible at the end of 2020. In general, vaccination is able to provide both health benefits for people, as well as to save medical care costs; reduce productivity loss; improve outcomes in unvaccinated community members (eg, through herd effects); and maintain economic, social, and political stability [5]. Vaccination may by some people even be perceived as a social contract that considers social welfare and moral obligation beyond self-interest, as a recent study found that the more compliant individuals regarding vaccination showed less generosity toward those who were not vaccinated [6].

On this background, vaccine hesitancy poses an issue for maximizing the population that could be vaccinated. It was found that one-third of the United Kingdom’s and Ireland’s population display COVID-19 vaccine hesitancy or even resistance [7]. Concerns about side effects and vaccine efficacy were two main reasons for not getting the COVID-19 vaccine [8]. Misinformation might worsen this situation, and in this regard, the provision of official COVID-19–related information in a comprehensible way could prevent people from seeking information or misinformation from nontrustworthy websites [9]. For example, YouTube has boosted the search rankings of provaccine videos, but viewers directed to antivaccine videos were likely to be exposed to additional links to antivaccine videos [10]. Therefore, governmental or official websites should serve as authoritative sources of COVID-19–related information. Readability and quality metrics of these websites are important, as the internet may eliminate barriers in the access to health information and hence eliminate misinformation for the public if the presented materials are of good quality and can be easily comprehended by the majority of the population [11]. Difficulty reading vaccine information may influence attitudes toward acceptance of or hesitancy to take vaccinations [12]. Previously, it was found that campaign websites could boost vaccine coverage [13]. The exact reasons were unclear, and it could have two implications. First, it could be that better websites would lead to higher vaccine coverage. Second, it could also be that places with lower vaccine coverage would put more effort to optimize their websites to promote/increase vaccine coverage. Regardless, it would be relevant to evaluate the relationship between website metrics and vaccination data.

This study aims to assess the readability and content quality of official COVID-19 vaccination campaign websites worldwide to reveal if their contents were easily understandable and mentioned the side effects and duration of vaccine protection, which are important factors in determining the support for getting vaccinated [14].

## Methods

### Data Source and Search Strategy

**Figure 1:**
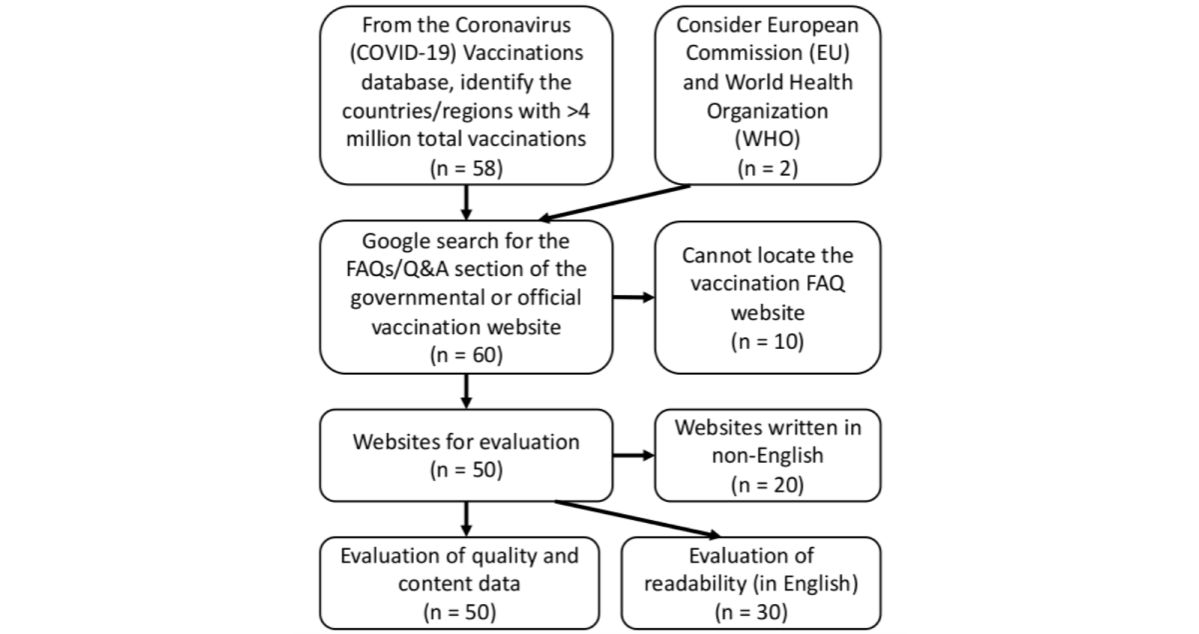
Flowchart of the selection of governmental or official COVID-19 vaccination websites for evaluation. FAQ: frequently asked question; Q&A: question and answer.

The open and actively updated data set named Coronavirus (COVID-19) Vaccinations [15], hosted by the Our World in Data database, was accessed on July 8, 2021. There were 58 countries/regions with their total vaccination count exceeding 4 million doses. For these 58 countries/regions the total vaccination count and vaccination dose per 100 people were noted. The governmental or official vaccination website was searched via Google with the phrases “official,” “COVID,” and “vaccine.” After entering these three terms, the first 10 result pages were examined to identify the relevant official vaccination website. The “frequently asked questions” (FAQs) or “questions and answers” (Q&As) section was identified and analyzed. This particular section was selected assuming that this would be the page where citizens sought for official information in the first place. Together with the websites of the World Health Organization (WHO) and European Union, we attempted to collect information from a total of 60 websites. As many European countries are in the European Union, the public living in Europe may look for information not only from their own country’s website but also from the EU website. Similarly, the WHO is the global health authority, and many people may look for information from its website as well. Therefore, websites from the WHO and European Union were included in this study. If there were multiple websites for one country (eg, vaccination websites from different governmental departments), then the one with FAQs targeting the public was selected. If such a section was not available from any of the websites, then the country would be coded as “cannot locate the vaccination FAQ website” (n=10; see Figure 1). The total vaccination count and vaccination dose per 100 people would be assessed with the website metrics to reveal if a better prepared website would relate to better vaccination coverage.

The website identification was performed by four authors (AWKY, EK, EDP, and AGA). The data extraction was performed by AWKY and double-checked by AGA. Disagreements in these procedures were solved by discussion and consensus reached between AWKY and AGA.

### Readability Assessment

For readability assessment (limited to websites with English versions only), the first 5 answers from the FAQs or Q&As page were transferred to Office Word software (Microsoft Corporation). The first 5 answers were used for evaluation, as online health information seekers rarely go beyond the first page of a search [16], and we reasoned that 5 FAQs would occupy a similar page space. The Flesch Reading Ease score and Flesch-Kincaid Grade Level statistic [17] were computed automatically by its built-in Spelling and Grammar function. A higher Flesch Reading Ease score indicates the text is more readable: reading difficulty is considered very easy if the score approaches 100, whereas the score range of 60 to 70 is considered standard [18,19]. In contrast, the value for the Flesch-Kincaid Grade Level indicates the grade level required for a reader to understand the text [17]. The American Medical Association and National Institutes of Health recommended that patient information materials should be written in the third to seventh grade level [20,21]. In the United States, the third grade means the third school year after kindergarten (third year of primary school) when students are aged around 8 to 9 years. Similarly, seventh grade means the seventh school year after kindergarten when students are aged around 12 to 13 years. The readability of websites without the English version were not evaluated, but they were still evaluated for the subsequent analyses (quality assessment and listing of protection duration and side effects) following Google Translation to English.

### Quality Assessment

Content quality was first assessed by the Health On the Net Foundation code (HONcode) certification status (ie, whether a website bore the HONcode badge). The Health on the Net Foundation is affiliated with the United Nations and issues the HONcode for health care websites that filed an application and met the required standard [22]. A dedicated web browser toolbar can be installed [23] to identify the certification status of a website, as used by previous researchers [24]. Having a HONcode certification is good, as it indicates the website reaches a standard of offering quality health information.

As this HONcode only gave a binarized certification status (certified or not) to the websites, we also used the Quality Evaluation Scoring Tool (QUEST) to evaluate the FAQ sections in seven aspects, namely, authorship, attribution to sources/references, type of study attributed to, conflict of interest, currency of presented information, complementarity of the patient-doctor relationship (support or no support of the relationship), and tone [25]. Readers can refer to the second figure by Robillard et al [25], published with open-access, for the complete description of the coding and scoring of QUEST. There is no cutoff score for QUEST, but the original research team reported that a score ≤11 indicated the worst scores from a survey of 290 online articles [26].

### Listing of Protection Duration and Side Effects

We recorded the number of Q&As from each website. The stated protection duration and the side effects of vaccinations listed on the websites were also recorded.

### Data Collection

It was not possible to collect all data within a single day. Data were collected since the day of the database search (July 8, 2021). On the last day of data collection (September 29, 2021), the publication dates and dates of the last update from all websites were collected to ensure a fairer evaluation of recency.

### Statistical Analysis

The Pearson correlation test was conducted to evaluate if the two readability metrics, QUEST score, and number of Q&As were correlated with the total vaccination count and vaccination dose per 100 people. Data from the WHO and European Union were not included in the correlation test or regional counts. Statistical tests were performed with SPSS (version 26.0; IBM Corp). Results were deemed significant if *P*<.05.

## Results

### Overall Data Collection Situation

The 60 investigated vaccination FAQs websites are listed in Table 1. By the United Nations geoscheme, 22 of the 58 selected countries/regions were in Asia, 22 in Europe, 4 in North America, 7 in South America, 2 in Africa, and 1 in Oceania. There were 10 countries for which we could not locate a vaccination FAQs website. There were 20 countries that provided non-English websites only. Therefore, quality and content data were available from 50 websites, whereas readability data were available from 30 websites.

**Table 1:** The 60 investigated governmental or official COVID-19 vaccination websites as accessed on July 8, 2021 (listed in descending order of total vaccination count).

Country/region	Total vaccination count (million), n	Vaccination dose per 100 people, n	Official vaccination FAQs^a^ website
China	1340.0	93.3	[27]
India	358.1	26.0	[28]
United States	331.7	99.2	[29]
Brazil	110.1	51.8	[30]
Germany	79.7	95.2	[31]
United Kingdom	79.5	117.2	[32]
France	57.2	84.6	[33]
Turkey	55.8	66.2	[34]
Italy	54.7	90.5	[35]
Japan	52.6	41.6	[36]
Mexico	48.5	37.6	[37]
Indonesia	48.5	17.7	[38]
Spain	45.2	96.8	[39]
Russia	45.1	30.9	[40]
Canada	40.7	107.8	[41]
Poland	30.7	81.2	[42]
Chile	23.6	123.2	[43]
Argentina	23.3	51.6	[44]
South Korea	19.9	38.8	[45]
Morocco	19.5	52.8	[46]
Colombia	19.4	38.2	Cannot locate the vaccination FAQs website
Saudi Arabia	19.1	54.8	[47]
Pakistan	18.2	8.3	[48]
Netherlands	16.9	98.6	[49]
United Arab Emirates	15.7	159.0	[50]
Philippines	12.2	11.1	[51]
Belgium	11.7	100.7	[52]
Thailand	11.6	16.7	[53]
Israel	10.9	125.7	[54]
Bangladesh	10.1	6.1	[55]
Malaysia	10.0	31.0	[56]
Hungary	9.8	101.9	Cannot locate the vaccination FAQs website
Portugal	9.5	93.0	[57]
Romania	9.1	47.1	[58]
Greece	9.0	86.0	[59]
Sweden	8.8	87.3	[60]
Czechia	8.7	81.2	[61]
Australia	8.6	33.6	[62]
Dominican Republic	8.4	77.4	Cannot locate the vaccination FAQs website
Peru	8.3	25.3	[63]
Austria	8.3	92.6	[64]
Cambodia	8.1	48.7	Cannot locate the vaccination FAQs website
Switzerland	7.7	89.1	[65]
Cuba	6.8	59.7	Cannot locate the vaccination FAQs website
Kazakhstan	6.1	32.7	[66]
Singapore	5.9	101.4	[67]
Iran	5.7	6.8	Cannot locate the vaccination FAQs website
Denmark	5.6	97.4	[68]
Serbia	5.3	78.2	Cannot locate the vaccination FAQs website
Ecuador	4.8	27.2	Cannot locate the vaccination FAQs website
Finland	4.6	82.8	[69]
Egypt	4.5	4.4	Cannot locate the vaccination FAQs website
Sri Lanka	4.5	21.2	Cannot locate the vaccination FAQs website
Ireland	4.5	90.9	[70]
Norway	4.4	81.4	[71]
Uruguay	4.2	121.3	[72]
Jordan	4.2	41.0	[73]
Hong Kong	4.1	55.3	[74]
World Health Organization	N/A^b^	N/A	[75]
European Commission	N/A	N/A	[76]

^a^FAQ: frequently asked question.

^b^N/A: not applicable.

### Readability Assessment

**Figure 2:**
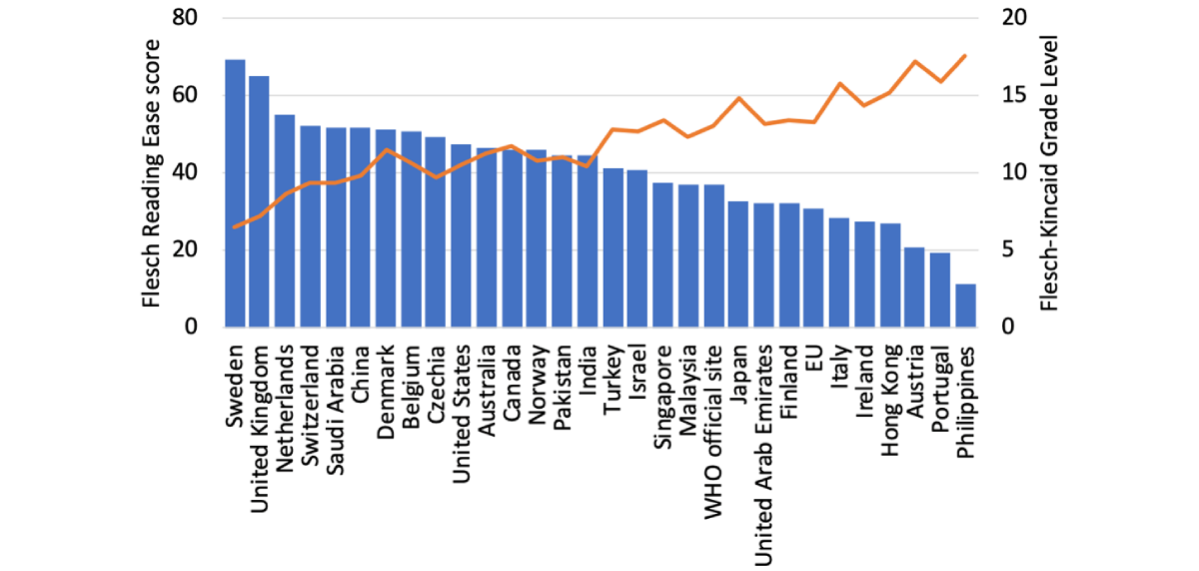
Readability assessment of the governmental or official COVID-19 vaccination websites. The recommended standards are >60 for Flesch Reading Ease score (blue bar) and <7 for Flesch-Kincaid Grade Level (orange line). WHO: World Health Organization.

The Flesch Reading Ease score of the vaccination FAQs websites ranged between 11.2 and 69.5, with a mean of 40.9 (SD 13.2). The websites of Sweden and the United Kingdom had a score above the threshold of 60 (Figure 2). In particular, the most difficult website was from the Philippines, with a score of 11.2.

Meanwhile, the Flesch-Kincaid Grade Level of the websites ranged between 6.5 to 17.6, with a mean of 12.1 (SD 2.8). Only the website of Sweden was within the targeted range (Flesch-Kincaid Grade Level 6.5; Figure 2).

### Quality Assessment

**Figure 3:**
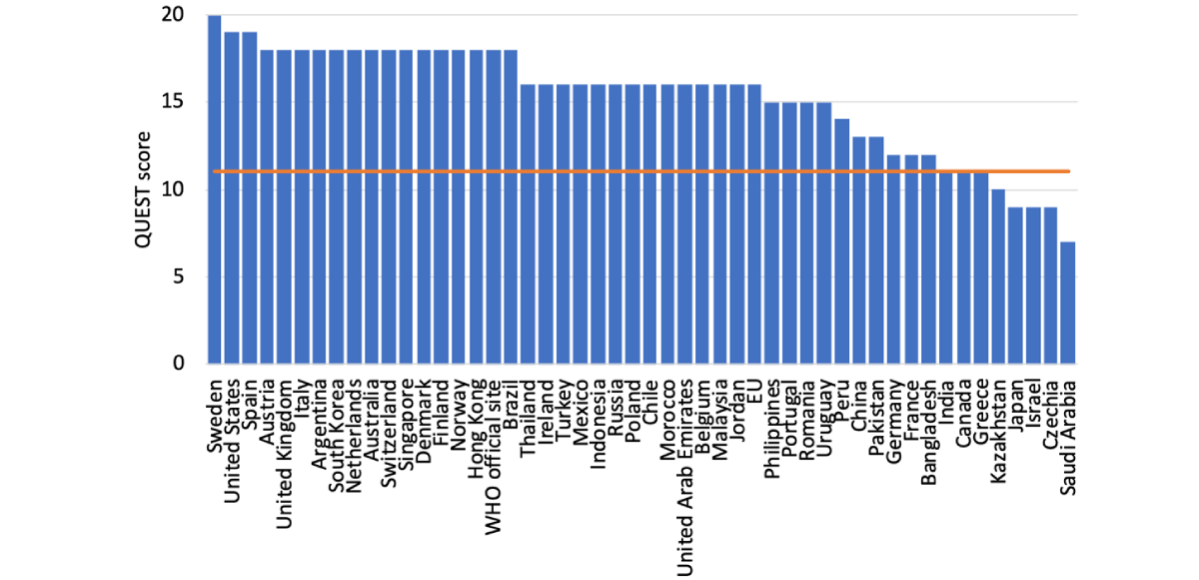
Quality assessment of the governmental or official COVID-19 vaccination websites. The recommended level of the QUEST score is ≥11 (orange line). QUEST: Quality Evaluation Scoring Tool; WHO: World Health Organization.

Only the websites from Switzerland and the WHO were HONcode certified. The QUEST score of the analyzed websites ranged between 7 and 20, with a mean of 15.3 (SD 3.1). Only 5 websites did not reach the recommended score of 11 (Figure 3). A detailed breakdown of the score revealed that only Sweden and Canada explicitly mentioned the authorship. None of the websites listed references to identifiable scientific studies. Besides, half of the websites (25/50) did not present a publication date or date of the last update. For the 25 websites that listed a date of the last update, all of them were updated in the year 2021 (n=12 in September, n=2 in August, n=3 in July, n=2 in June, n=3 in April, n=2 in February, and n=1 in January). Four of them also listed a publication date, ranging from October 23, 2020, to April 7, 2021.

### Listing of Protection Duration and Side Effects

**Figure 4:**
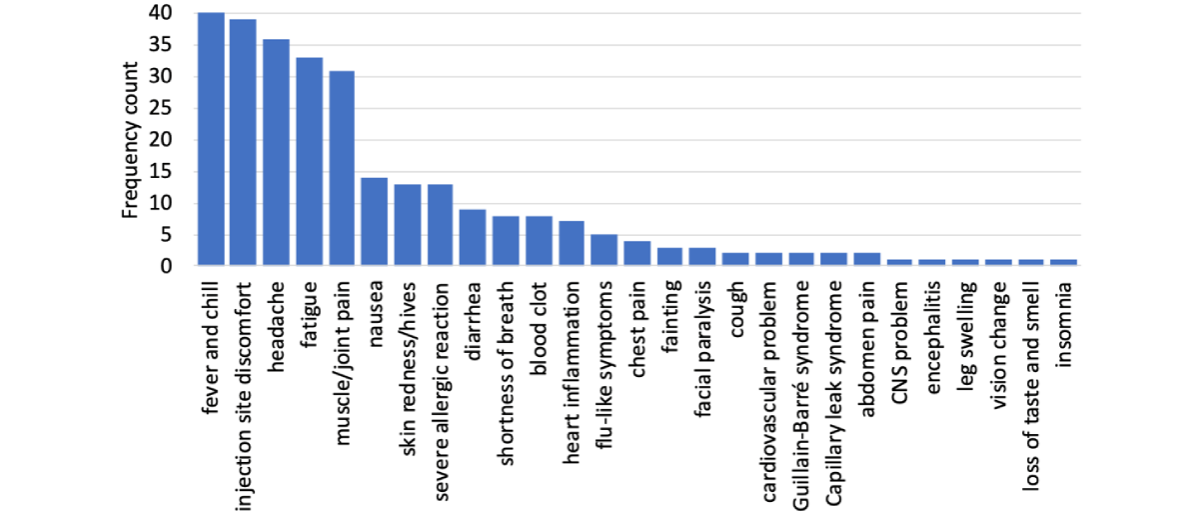
List of side effects caused by vaccination as mentioned by the governmental or official COVID-19 vaccination websites. CNS: central nervous system.

The number of Q&As listed in the websites ranged from 6 to 150, with a mean of 36.5 (SD 35.1). Regarding the duration of protection offered by the vaccines, 46% (23/50) of the websites stated that they do not know, and another 40% (n=20) did not address this aspect. The remaining websites stated that the vaccine could provide protection for at least 6 months (n=2), 6 to 8 months (n=1), at least 9 months (n=1), at least 9 to 12 months (n=1), and at least 12 months (n=2). Five side effects were most frequently mentioned, namely, fever/chill (n=41, 82%), various injection site discomfort events (eg, swelling, redness, or pain; n=39, 78%), headache (n=36, 72%), fatigue (n=33, 66%), and muscle/joint pain (n=31, 62%). Other side effects were mentioned much less frequently (<28%). Figure 4 lists the frequency counts of mentioned side effects.

### No Relationship Between Vaccination and Website Metrics

Pearson correlation tests revealed that the Flesch Reading Ease score, Flesch-Kincaid Grade Level, QUEST score, and number of Q&As had no significant relationship with total vaccination count (*P*=.35, .29, .47, and .23, respectively) and vaccination dose per 100 people (*P*=.30, .44, .33, and .59, respectively).

## Discussion

### Principal Findings

As information on COVID-19 vaccination might influence vaccination progress, we set out to assess governmental websites providing information on COVID-19 vaccination in 60 countries/regions and organizations, and finally identified and analyzed 50 websites that promote COVID-19 vaccination programs.

Readability remained an issue for most of the analyzed websites. Even though we analyzed the FAQs section of the websites, which are supposed to be reader friendly, all but 1 failed to meet the recommendation by the American Medical Association and National Institutes of Health of being easily understood by a seventh grader or below [20,21], and only 2 met the recommendation of a Flesch Reading Ease score of 60 to 70 [18,19]. One of the potential reasons could be that the information disseminated in these vaccination campaign websites must be accurate and explanatory to minimize any risks of legal consequences. Hence, the choice of words would be careful and explanatory clauses added to lengthen the sentences. For example, the Philippines website was as difficult to read as a standard auto insurance policy (see Flesch [77] for more examples). It listed a simple question “are there enough vaccines for filipinos” with an answer composed of 2 long sentences with many polysyllabic words:

The prioritization framework for COVID-19 vaccination based on the WHO Strategic Advisory Group of Experts (SAGE) on Immunization, together with the recommendations of independent bodies of experts including the Interim National Immunization Technical Advisory Group (iNITAG) and the Technical Advisory Group (TAG), was formulated due to the limited global supply of COVID-19 vaccine products. With the guidance of this principle, the Philippine National Deployment and Vaccination Plan formulated strategies and contingencies to ensure the equitable distribution of vaccine products for all Filipinos.

Certainly, readability and content quality are two different aspects valued by online health information seekers. HONcode certification was reported as an indicator correlated to the content accuracy of online health information [78]. In this context, it is interesting to observe that the surveyed vaccination websites did not present certifications. Since the Health On the Net Foundation is affiliated with the United Nations and its certification is globally recognized, more efforts should be made to get certified, especially as these vaccination websites are accessed by many people on a daily and global basis. However, most of them actually had a QUEST score of ≥11, meaning that their content quality was good. The most frequently listed side effects in the websites were consistent to the ones most frequently reported by people receiving the COVID-19 vaccines [79,80]. This consistency is beneficial, as information seekers will feel that the website is trustworthy and provides accurate descriptions of what to be expected if they want to be vaccinated. Although none of the websites listed references to identifiable scientific studies, it could be argued that the FAQs websites were primarily intended for provision of the most important and updated information; references might not be highly sought after by the information seekers in this scenario.

The majority of the websites either did not mention the duration of protection or stated that they did not know how long vaccination could protect citizens. Despite this particular missing (or unknown) piece of information, these 48 countries/regions still had the highest total vaccination counts. To get vaccinated may be influenced by many different considerations, wherefore readability, quality, and content of the vaccination websites were not the only potential influencing factors. Higher frequency of exposure to positive information about COVID-19 vaccination was associated with a higher intention to be vaccinated [81]. Meanwhile, public health authorities should also monitor social media continuously to identify newly developed antivaccine arguments and provide updated information to clear the doubts and debunk the myths [82]. These aspects should be carefully considered by the authorities as they update/improve the campaign websites. This survey found that only a small proportion of the websites were last updated in September 2021. It suggested that the rest of these websites should be updated more frequently to provide up-to-date information.

Initially, it was a bit surprising to find that there was no relationship between vaccination counts and website metrics. However, it could be explained by the fact that people could access vaccine information via many sources such as newspapers, magazines, YouTube, and Facebook. A previous study [83] revealed that the commonest reasons for agreeing to be vaccinated included “to protect themselves and others” (29%), “belief in vaccination and science” (16%), and “to help stop the virus spread” (15%), which could be intuitive and might not be affected by vaccination website readability.

This study found that only 2 of the 50 surveyed websites were HONcode certified. This was consistent to a recent survey on online misinformation about COVID-19, for which the certification rate was reported to be 1.8% (2/110) [84]. It was similarly reported that it was difficult to comprehend the privacy policies of COVID-19 contact tracing apps, with their reading level between 7th and 14th grade [85]. Considered together, the take-home message for the authoritative bodies and policy makers is to further improve the COVID-19 vaccination campaign items (eg, websites or apps) by making them more comprehensible and more trustworthy with certification.

This study surveyed the FAQs section of official vaccination websites. However, people may seek online information from other sources such as online discussion forums, news outlets, and blogs. Readability and content quality of such sources was outside the scope of this study and might provide an interesting topic for further research. Furthermore, some official vaccination websites did not have FAQs sections, and thus, no data were extracted. Meanwhile, the initial decision to target 60 websites/countries was relatively arbitrary. Therefore, readers should be aware that not all websites across all countries were included. For the non-English language websites, content were only analyzed after translation to English via Google Translate. Therefore, readability could not be assessed. Even for the websites that presented English materials, some biases or issues may still exist. In particular, because English could be a primary language for some countries but not the others or even for certain populations within a country instead of the entire population, bias might be introduced. Hence, the content could be written as a primary version, a translated version, or even provided by automated website translation beyond our notice. Instead of choosing the desired language by clicking a button, a country might provide FAQs sections in other languages at completely different websites not evaluated in this study. In addition, the potential reuse of content from EU/WHO websites by country websites was not assessed. Readers should be aware of these issues during the interpretation of the readability assessment results.

### Conclusions

The following points could be concluded: the content quality was good, HONcode certification should be considered, content should be written in a more readable manner, and web pages should be updated more frequently to keep the information up to date. In short, COVID-19 vaccination FAQs websites basically provide good quality information, but more efforts should be paid to make them more readable and updated.

## Abbreviations

FAQ: frequently asked question
HONcode: Health On the Net Foundation code
QUEST: Quality Evaluation Scoring Tool
Q&A: question and answer
WHO: World Health Organization

## References

[1] Onder G, Rezza G, Brusaferro S (2020). Case-fatality rate and characteristics of patients dying in relation to COVID-19 in Italy. JAMA.

[2] Meyerowitz-Katz G, Merone L (2020). A systematic review and meta-analysis of published research data on COVID-19 infection fatality rates. Int J Infect Dis.

[3] Ghaffari Darab M, Keshavarz K, Sadeghi E, Shahmohamadi J, Kavosi Z (2021). The economic burden of coronavirus disease 2019 (COVID-19): evidence from Iran. BMC Health Serv Res.

[4] Di Fusco M, Shea KM, Lin J, Nguyen JL, Angulo FJ, Benigno M, Malhotra D, Emir B, Sung AH, Hammond JL, Stoychev S, Charos A (2021). Health outcomes and economic burden of hospitalized COVID-19 patients in the United States. J Med Econ.

[5] Bärnighausen T, Bloom DE, Cafiero-Fonseca ET, O'Brien JC (2014). Valuing vaccination. Proc Natl Acad Sci U S A.

[6] Korn L, Böhm R, Meier NW, Betsch C (2020). Vaccination as a social contract. Proc Natl Acad Sci U S A.

[7] Murphy J, Vallières F, Bentall RP, Shevlin M, McBride O, Hartman TK, McKay R, Bennett K, Mason L, Gibson-Miller J, Levita L, Martinez AP, Stocks TVA, Karatzias T, Hyland P (2021). Psychological characteristics associated with COVID-19 vaccine hesitancy and resistance in Ireland and the United Kingdom. Nat Commun.

[8] Solís Arce JS, Warren SS, Meriggi NF, Scacco A, McMurry N, Voors M, Syunyaev G, Malik AA, Aboutajdine S, Adeojo O, Anigo D, Armand A, Asad S, Atyera M, Augsburg B, Awasthi M, Ayesiga GE, Bancalari A, Björkman Nyqvist M, Borisova E, Bosancianu CM, Cabra García MR, Cheema A, Collins E, Cuccaro F, Farooqi AZ, Fatima T, Fracchia M, Galindo Soria ML, Guariso A, Hasanain A, Jaramillo S, Kallon S, Kamwesigye A, Kharel A, Kreps S, Levine M, Littman R, Malik M, Manirabaruta G, Mfura JLH, Momoh F, Mucauque A, Mussa I, Nsabimana JA, Obara I, Otálora MJ, Ouédraogo BW, Pare TB, Platas MR, Polanco L, Qureshi JA, Raheem M, Ramakrishna V, Rendrá I, Shah T, Shaked SE, Shapiro JN, Svensson J, Tariq A, Tchibozo AM, Tiwana HA, Trivedi B, Vernot C, Vicente PC, Weissinger LB, Zafar B, Zhang B, Karlan D, Callen M, Teachout M, Humphreys M, Mobarak AM, Omer SB (2021). COVID-19 vaccine acceptance and hesitancy in low- and middle-income countries. Nat Med.

[9] Kricorian K, Civen R, Equils O (2021). COVID-19 vaccine hesitancy: misinformation and perceptions of vaccine safety. Hum Vaccin Immunother.

[10] Tang L, Fujimoto K, Amith M, Cunningham R, Costantini RA, York F, Xiong G, Boom JA, Tao C (2021). "Down the Rabbit Hole" of vaccine misinformation on YouTube: network exposure study. J Med Internet Res.

[11] Berland GK, Elliott MN, Morales LS, Algazy JI, Kravitz RL, Broder MS, Kanouse DE, Muñoz JA, Puyol J, Lara M, Watkins KE, Yang H, McGlynn EA (2001). Health information on the Internet: accessibility, quality, and readability in English and Spanish. JAMA.

[12] Okuhara T, Ishikawa H, Ueno H, Okada H, Kato M, Kiuchi T (2022). Readability assessment of vaccine information: a systematic review for addressing vaccine hesitancy. Patient Educ Couns.

[13] Odone A, Ferrari A, Spagnoli F, Visciarelli S, Shefer A, Pasquarella C, Signorelli C (2015). Effectiveness of interventions that apply new media to improve vaccine uptake and vaccine coverage. Hum Vaccin Immunother.

[14] Kreps S, Prasad S, Brownstein JS, Hswen Y, Garibaldi BT, Zhang B, Kriner DL (2020). Factors associated with US adults' likelihood of accepting COVID-19 vaccination. JAMA Netw Open.

[15] Mathieu E, Ritchie H, Ortiz-Ospina E, Roser M, Hasell J, Appel C, Giattino C, Rodés-Guirao L (2021). A global database of COVID-19 vaccinations. Nat Hum Behav.

[16] Morahan-Martin JM (2004). How internet users find, evaluate, and use online health information: a cross-cultural review. Cyberpsychol Behav.

[17] Stockmeyer NO (2009). Using Microsoft Word's readability program. Michigan Bar J.

[18] D'Alessandro DM, Kingsley P, Johnson-West J (2001). The readability of pediatric patient education materials on the World Wide Web. Arch Pediatr Adolesc Med.

[19] Finn S (2016). Unpredictability as correlate of reader enjoyment of news articles. Journalism Q.

[20] Prabhu AV, Donovan AL, Crihalmeanu T, Hansberry DR, Agarwal N, Beriwal S, Kale H, Heller M (2018). Radiology online patient education materials provided by major university hospitals: do they conform to NIH and AMA guidelines?. Curr Probl Diagn Radiol.

[21] Prabhu AV, Hansberry DR, Agarwal N, Clump DA, Heron DE (2016). Radiation oncology and online patient education materials: deviating from NIH and AMA recommendations. Int J Radiat Oncol Biol Phys.

[22] Boyer C, Baujard V, Geissbuhler A (2011). Evolution of health web certification through the HONcode experience. Stud Health Technol Inform.

[23] Tools. Health On the Net.

[24] Schwarzbach HL, Mady LJ, Kaffenberger TM, Duvvuri U, Jabbour N (2021). Quality and readability assessment of websites on human papillomavirus and oropharyngeal cancer. Laryngoscope.

[25] Robillard JM, Jun JH, Lai J, Feng TL (2018). The QUEST for quality online health information: validation of a short quantitative tool. BMC Med Inform Decis Mak.

[26] Robillard JM, Feng TL (2017). Health advice in a digital world: quality and content of online information about the prevention of Alzheimer's disease. J Alzheimers Dis.

[27] Common questions about coronavirus vaccinations. National Health Commission of the People's Republic of China.

[28] FAQs on COVID-19 vaccine. Ministry of Health and Family Welfare, Government of India.

[29] Frequently asked questions about COVID-19 vaccination. Centers for Disease Control and Prevention.

[30] Perguntas e respostas. Governo do Brasil.

[31] Aktuelle informationen zur COVID-19-Impfung. Bundesministerium für Gesundheit.

[32] Coronavirus (COVID-19) vaccination. NHS.

[33] FAQ Covid-19. Santé.fr.

[34] Frequently asked questions. T.C. Sağlık Bakanlığı.

[35] FAQ - COVID-19 vaccines. Italian Medicines Agency.

[36] COVID-19 vaccines. Prime Minister of Japan and His Cabinet.

[37] Preguntas frecuentes. Vacuna Covid.

[38] Tanya jawab. Covid19.go.id.

[39] Vacunación COVID-19 preguntas comunes. Ministerio de Sanidad.

[40] Ответы на самые распространенные вопросы о вакцинах и вакцинации. коронавирусе COVID-19: вакцина.

[41] Ask the experts COVID-19 vaccines questions: safety, ingredients and side effects. Canada.ca.

[42] Pytania i odpowiedzi. Gov.pl.

[43] Preguntas frecuentes. Gob.cl.

[44] Preguntas frecuentes sobre la vacuna contra COVID-19. Argentina.gob.ar.

[45] 코로나19 백신. 코로나19예방접종.

[46] Les réponses aux questions fréquentes posées autour de la vaccination contre la COVID-19. Campagne de vaccination contre le coronavirus au Maroc.

[47] Ministry of Health.

[48] COVID vaccination. National Command Operation Center.

[49] Getting vaccinated against COVID-19 in the Netherlands (translations). Government of the Netherlands.

[50] About the vaccine. UAE Coronavirus (COVID-19) Updates.

[51] #ChecktheFAQs: a campaign to fight vaccine misinformation!. Department of Health, Republic of the Philippines.

[52] Frequently asked questions (FAQs). Coronavirus Covid-19.

[53] Thailand's Covid-19 vaccine. Department of Disease Control.

[54] COVID-19 questions and answers. gov.il.

[55] Commonly asked. corona.gov.bd.

[56] Popular questions. vaksincovid.gov.my.

[57] Vaccination | common questions. SNS24.

[58] Întrebări și răspunsuri. Guvernul Romaniei.

[59] Ερωτήσεις / Απαντήσεις για τον Εμβολιασμό του πληθυσμού απέναντι στον Covid-19. Αρχική Εμβολιασμός COVID-19.

[60] Vaccination against COVID-19. 1177 Vårdguiden.

[61] Questions and answers about vaccination. Ministry of Health of Czech Republic.

[62] Is it true? Get the facts on COVID-19 vaccines. Australian Government Department of Health.

[63] Mitos sobre las vacunas contra la COVID-19. Gobierno del Perú.

[64] COVID-19 vaccination. Bundesministerium für Soziales, Gesundheit, Pflege und Konsumentenschutz.

[65] Coronavirus: frequently asked questions (FAQs). Bundesamt für Gesundheit BAG.

[66] Что нужно знать о вакцине от COVID-19. Corona Virus 2020 KZ.

[67] FAQS - safety and efficacy of the COVID-19 vaccine. Ministry of Health Singapore.

[68] Vaccination against COVID-19. Sundhedsstyrelsen.

[69] Safety of COVID-19 vaccines. Terveyden ja hyvinvoinnin laitos.

[70] COVID-19 vaccines: frequently asked questions. The Health Products Regulatory Agency.

[71] Coronavirus vaccine - information for the public. Folkehelseinstituttet.

[72] Preguntas frecuentes vacunación COVID-19. Sitio oficial de la República Oriental del Uruguay.

[73] سؤال وجواب. corona.moh.gov.jo.

[74] FAQs. The Government of the Hong Kong Special Administration Region.

[75] Coronavirus disease (COVID-19): vaccines safety. World Health Organization.

[76] COVID-19 vaccines: key facts. European Medicines Agency.

[77] Flesch R (1979). How to Write Plain English: A Book for Lawyers and Consumers.

[78] Fallis D, Frické M (2002). Indicators of accuracy of consumer health information on the Internet: a study of indicators relating to information for managing fever in children in the home. J Am Med Inform Assoc.

[79] Klugar M, Riad A, Mekhemar M, Conrad J, Buchbender M, Howaldt H, Attia S (2021). Side effects of mRNA-based and viral vector-based COVID-19 vaccines among German healthcare workers. Biology (Basel).

[80] Riad A, Pokorná A, Attia S, Klugarová J, Koščík M, Klugar M (2021). Prevalence of COVID-19 vaccine side effects among healthcare workers in the Czech Republic. J Clin Med.

[81] Zhang KC, Fang Y, Cao H, Chen H, Hu T, Chen Y, Zhou X, Wang Z (2021). Behavioral intention to receive a COVID-19 vaccination among Chinese factory workers: cross-sectional online survey. J Med Internet Res.

[82] Wawrzuta D, Jaworski M, Gotlib J, Panczyk M (2021). Characteristics of antivaccine messages on social media: systematic review. J Med Internet Res.

[83] Dodd RH, Pickles K, Nickel B, Cvejic E, Ayre J, Batcup C, Bonner C, Copp T, Cornell S, Dakin T, Isautier J, McCaffery KJ (2021). Concerns and motivations about COVID-19 vaccination. Lancet Infect Dis.

[84] Cuan-Baltazar JY, Muñoz-Perez MJ, Robledo-Vega C, Pérez-Zepeda MF, Soto-Vega E (2020). Misinformation of COVID-19 on the internet: infodemiology study. JMIR Public Health Surveill.

[85] Zhang M, Chow A, Smith H (2020). COVID-19 contact-tracing apps: analysis of the readability of privacy policies. J Med Internet Res.

